# Removal of two cytostatic drugs: bleomycin and vincristine by white-rot fungi – a sorption study

**DOI:** 10.1007/s40201-021-00635-8

**Published:** 2021-03-05

**Authors:** Marcelina Jureczko, Wioletta Przystaś

**Affiliations:** 1 Environmental Biotechnology Department, Faculty of Energy and Environmental Engineering, The Silesian University of Technology, Akademicka 2, 44-100 Gliwice, Poland; 2 The Biotechnology Centre, The Silesian University of Technology, Krzywoustego 8, 44-100 Gliwice, Poland

**Keywords:** Anticancer drugs, Aquatic systems, Biosorption, Cytostatics, Fungi

## Abstract

**Purpose:**

Cytostatic drugs cannot be easily removed by conventional sewage treatment plants, resulting in their ultimate release into aquatic systems where they become a threat. Thus, new technologies which can be used to eliminate these drugs more effectively before they enter the environment are increasingly important. Fungal treatment of wastewaters is a promising and environmentally friendly technology for pharmaceutical remediation. The aim of this work is to examine the biosorption of two cytostatics, bleomycin and vincristine, in the aqueous solution by fungal biomass.

**Methods:**

Five white-rot fungi were used in this study: *Fomes fomentarius* (CB13), *Hypholoma fasciculare* (CB15), *Phyllotopsis nidulans* (CB14), *Pleurotus ostreatus* (BWPH), and *Trametes versicolor* (CB8). Tests were conducted on different types of biomass (alive and dead – autoclaved) and in various physico-chemical conditions: varied drug concentrations (5, 10 and 15 mg/L), temperatures (from 15.4 to 29.6 °C), and pH (from 3.2 to 8.8).

**Results:**

The results showed that among alive biomass, *T. versicolor* (CB8) had the greatest sorption ability for bleomycin and *P. nidulans* (CB14) worked best for vincristine. The tested sorption process could be described by a pseudo-second order kinetics model. Sorption equilibrium studies demonstrated that for bleomycin Redlich-Peterson, while for vincristine Langmuir model fitted best. The thermodynamic studies showed that the sorption process was endothermic chemisorption for bleomycin, and exothermic physisorption for vincristine. For both drugs the sorption ability increased with an increase of the pH value.

**Conclusion:**

The biosorption on fungal biomass is a favorable alternative to conventional wastewater treatment processes for anticancer drug removal.

## Introduction

The consumption of pharmacologically active compounds (PhACs) has been steadily increasing, reaching thousands of tons annually [9, 25, 35]. Unfortunately, many of these chemicals cannot be easily removed by conventional sewage treatment plants (STPs) due to their hydrophilic character, persistent nature, and their relatively low concentrations in wastewater [35, 51, 55, 61]. During the removal process, one of the most problematic groups are compounds with an aromatic structure, which makes them less susceptible to biodegradation. Among these, cytostatic drugs, worldly used as cancer remedy, warrant particular attention [6, 20, 64]. These substances, comprised of synthetic and natural compounds, are primarily used to inhibit or completely block the replication of deoxyribonucleic acid in tumour cells, which can lead to cell death [6, 16, 20, 41, 48, 64]. Much research has shown that unmetabolised cytostatic drugs, as well as their transformation products derived from hospital effluents, municipal wastewater, and drug manufacturers, not only occur in sewage and STP effluent, but also have been detected in surface, ground, and drinking waters [9, 16, 33, 36–38, 45, 50, 51, 55, 56, 63, 64]. Unfortunately, cytostatic drugs are not cancer-specific and can impact the stability of the genetic material in aquatic organisms [36, 64]. As such they are considered to be emerging pollutants [9, 55, 56]. Even though the risk of acute toxic effects in fauna as well as flora is unlikely, the possibility of chronic environmental toxic effects cannot be excluded [9]. In addition, ground water and surface water are frequently used as sources of potable water. Thus there is the potential for adverse effects on human health [48, 58].

In this study, we focused on two cytostatics, widely used in the cancer therapy, and never thoroughly studied before: bleomycin and vincristine. Concentrations of these pharmaceuticals in hospital effluent approach 124,000 ng/L and 50 ng/L, respectively [8, 44, 53]. Both drugs are found in concentrations of up to approximately 20 ng/L in WWTP (wastewater treatment plant) influent as well as effluent, and have also been detected in rivers and drinking water [2, 30, 53, 62]. According to Jureczko and Przystaś [26], ecotoxicological tests have classified bleomycin as very toxic, while vincristine is considered a toxic water pollutant under EU-Directive 93/67/ EEC. Therefore, new technologies which can be used to eliminate these cytostatics from the environment are increasingly a focus of research [35]. For pharmaceutical remediation processes, the fungal treatment of wastewaters has been highlighted as an environmentally friendly and potentially promising technology [24, 34, 35]. White-rot fungi (WRF) have shown great potential in the removal of PhAC compounds due to the synergistic effects of an unspecific multi-enzyme (both extracellular and intercellular) system [3, 10, 11, 31, 46, 49, 61]. The biodegradation mechanisms of living cells is of high importance, the contribution of sorption to the overall elimination of pharmaceuticals by fungi cannot be neglected [4, 24, 34, 35, 39]. This physico-chemical process is competitive, effective, energy-independent, fast, reversible, and can be conducted both on activated and inactivated biomass [24, 34, 35, 39, 54]. This process works both through absorption and adsorption (former consisting in the entry of pollutants into the biomass, while the latter in the adhesion of pollutants to the biomass surface) [35, 39]. Macro fungal biomass is an ideal biosorbent, due to its easy availability and a relatively low cost, as it can be produced using simple fermentation techniques on cheap growth media or can be obtained as a by-product of various industrial processes [42]. Using the sorption process on WRF biomass is a popular technique tested for the removal of other pollutants that are not easily biodegraded, such as dyes, heavy metals, personal care products, PAHs (polycyclic aromatic hydrocarbons), and odour causing substances [12, 19, 21, 23, 35, 54]. Among treatment techniques, sorbent usage has been found to be superior to others with the reference to the ease of operation, flexibility and simplicity of design, as well as insensitivity to toxic pollutants [54]. Finally, and importantly, the formation of harmful substances is not the result of the sorption process [54].

Even though fungal treatment of wastewater has been pointed out as a promising and environmentally friendly technology for pharmaceutical remediation processes, there exist extremely few articles about this method of cytostatics removal. In addition, it is worth emphasizing that research on biosorption has never been conducted. Thus, the objective of the current study was to examine the biosorption of bleomycin and vincristine to fungal biomass, and to establish the relative contributions of sorption in the total removal of these drugs by WRF. The cytostatics chosen for this study had never been thoroughly studied. Furthermore, not only did we focus on two strains of the most promising fungal species, *Trametes versicolor* and *Pleurotus ostreatus,* for which good PhACs removal values had already been confirmed, but we also sought to find new candidates by analysing the cytostatics sorption removal efficiency of *Fomes fomentarius, Hypholoma fasciculare,* and *Phyllotopsis nidulans.*

## Materials and methods

### Test compounds and solutions preparation

Bleomycin sulphate (CAS: 9041-93-4) and vincristine sulphate (CAS: 2068-78-2) (Chemat, Gdańsk, Poland) were used to prepare concentrated solutions (2500 mg/L) in deionized water. These solutions were further used to prepare tests solutions, by mixing appropriate volumes of the stock solutions with deionized water.

### Fungi species isolation and identification

In test were used: *Fomes fomentarius* (CB13), *Hypholoma fasciculare* (CB15), *Phyllotopsis nidulans* (CB14), *Pleurotus ostreatus* (BWPH) and *Trametes versicolor* (CB8), which are deposited in Fungal Strain Collection of The Biotechnology Centre, The Silesian University of Technology, Gliwice, Poland. Their identification confirmed using molecular biology methods was previously described in Jureczko et al. [27] work. Fungal biomass was cultivated in liquid medium (2 × diluted Nutrient Broth N° 1, Fluka) at 22.5 °C for 30 days before test. To prepare alive fungal biomass, mycelia were washed with sterile deionized water for 24 h before its use, while dead one was rinsed in water and autoclaved (at 121 °C for 15 min).

### Sorption tests

Each strain’s ability to eliminate selected anticancer drugs by sorption was tested by placing 0.1 g of fungal biomass (both alive – rinsed for 24 h in water, and dead - autoclaved) in vials containing 10 ml of cytostatics aqueous solutions (in sterile deionised water) at a concentration of 10 mg/L. The process was carried out for 4 h at 22.5 °C. The natural pH of bleomycin sulphate and vincristine sulphate water solutions were 4.5 and 3.6, respectively. The reduction in drug concentration was checked at the following points: 0 min, 15 min, 30 min, 1 h, 2 h, 3 h, and 4 h. Sorption evaluation was done indirectly by measuring the pollutant concentration in solution. The amount of adsorbed drugs was calculated as a difference before and after adsorption with correction for the loss observed in the abiotic sample (control). The concentration of cytostatic drugs in samples was determined using UV-ViS spectrophotometer (Hitachi U-1900) at 210 nm for bleomycin and 219 nm for vincristine. Standard curves were prepared in the range of 1–20 mg/L. Blanks containing no cytostatics were used for each series of experiments. All the assays were conducted in at least three experimental replicates. This experiment allowed for a test of the effect of contact time and type of biomass. In parallel, the dry matter content after drying for 7 days in 35 °C was measured. These values were used to determine the removal by gram of dry matter of the mycelium.

Further tests were conducted using alive biomass for the two fungi which demonstrated the best ability to remove each drug. The effect of initial anticancer drug dose was investigated with three different cytostatic concentrations: 5 mg/L, 10 mg/L, 15 mg/L. The influence of temperature (from 15.4 to 29.6 °C) and pH (from 3.2 to 8.8, adjusted with 0.1 M NaOH and HCl) were tested together using Central Composite Design (CCD). The concentration of cytostatics in each sample was measured after 3 h of contact time according to sorption kinetics. CCD, which could be followed by response surface methodology (RSM), expresses the mathematical relation between influence of independent and interactive influence of measured parameters and is approximated by a polynomial quadratic formula (Eq. 1). It consists of 12 experimental sets, with 9 experimental set-ups for k = 2 factor analysis, and four replications in the center point to obtain higher experimental precision. For the first-order regression coefficients, a center point is required, as well as k^2^ factorial points. For estimations in the second-order model 2 k axial points are needed. Because the distance from the center point to the factorial as well as axial points is the same and equals α = √k, this gives a spherical and rotatable design [59, 60].

1 $$ \mathrm{Effect}=a\ \mathrm{p}\mathrm{H}+b\ \mathrm{T}+c\ \mathrm{p}{\mathrm{H}}^2+d\ {\mathrm{T}}^2+e\ \mathrm{p}\mathrm{H}\ \mathrm{T}+f $$

where T is temperature, and a, b, c, d, e, f are coefficients.

### Data analysis, adsorption kinetics, biosorption isotherms and thermodynamic parameters

The percentage of drugs removed was calculated using the following formula:

2 $$ \mathrm{Removal}\ \left(\%\right)=\frac{{\mathrm{C}}_{\mathrm{i}}-{\mathrm{C}}_{\mathrm{f}}}{{\mathrm{C}}_{\mathrm{i}}}\ \mathrm{x}\ 100 $$

where C_i_ is the initial drug concentration and C_f_ is the final drug concentration at the end of experiment in mg/L.

The maximum amount of drug adsorption at measurement points was determined using the following equation:

3 $$ {\mathrm{q}}_{\mathrm{t}}=\left({\mathrm{C}}_{\mathrm{i}}-{\mathrm{C}}_{\mathrm{t}}\right)\frac{\mathrm{V}}{\mathrm{M}} $$

where C_i_ is the initial drug concentration and C_t_ is the concentration at time t (mg/L), V is the solution volume (L), and M is the mass of fungi biomass used (g) [21].

The q_e_ – the maximum amount of drug adsorption at equilibrium, is read from plot q_t_ vs time, at particular time when q values saturates.

Adsorption kinetics was performed using pseudo first- and second-order kinetics, as well as Elovich kinetic using the following expressions:

4 $$ \mathrm{Pseudo}\ \mathrm{first}\ \mathrm{order}\ \mathrm{equation}:\log \left({\mathrm{q}}_{\mathrm{e}}-{\mathrm{q}}_{\mathrm{t}}\right)=\log \left({\mathrm{q}}_{\mathrm{e}}\right)-\left(\frac{{\mathrm{k}}_1}{2.303}\right)\mathrm{t} $$

5 $$ \mathrm{Pseudo}\ \mathrm{second}\ \mathrm{order}\ \mathrm{equation}:\frac{\mathrm{t}}{{\mathrm{q}}_{\mathrm{t}}}=\left(\frac{1}{{\mathrm{k}}_2\ {\mathrm{q}}_{\mathrm{e}}^2}\right)+\left(\frac{1}{{\mathrm{q}}_{\mathrm{e}}}\right)\mathrm{t} $$

6 $$ \mathrm{Elovich}\ \mathrm{linear}\ \mathrm{form}\ \mathrm{of}\ \mathrm{equation}:{\mathrm{q}}_{\mathrm{t}}=\frac{\ln \upalpha \mathrm{b}}{\mathrm{b}}+\frac{1}{\mathrm{b}}\ln \mathrm{t} $$

where q_e_ is the amount of drug adsorbed at equilibrium (mg/g), q_t_ is the amount of drug adsorbed at time t (mg/g), k_1_ (1/min) is the pseudo first-order rate constant of the equation calculated from the slope of the plot log(q_e_ − q_t_) vs. t, and k_2_ (g/mg·min) is the pseudo second-order rate constant. A plot of t/q_t_ vs. t yields a straight line with a slope of 1/q_e_. The value of k_2_ is determined from the intercept of the plot [7]. In the Elovich model the parameter α is the initial rate of adsorption (mg/g·min) and b is related to the extent of surface coverage and activation energy for chemisorption (g/mg). Parameters α and b were determined using the slope and intercept from plot q_t_ against ln t [15, 52].

The experimental data for drug removal were tested with Langmuir, Freundlich and Redlich-Peterson isotherms. The linear form of this model’s equation is represented by the following expressions:

7 $$ \mathrm{Langmuir}:\frac{1}{{\mathrm{q}}_{\mathrm{e}}}=\frac{1}{{\mathrm{q}}_0}+\frac{1}{{\mathrm{q}}_0{\mathrm{K}}_{\mathrm{L}}{\mathrm{C}}_{\mathrm{e}}} $$

8 $$ \mathrm{Freundlich}:\log\ {\mathrm{q}}_{\mathrm{e}}=\log\ {\mathrm{K}}_{\mathrm{f}}+\frac{\log\ {\mathrm{C}}_{\mathrm{e}}}{\mathrm{n}} $$

9 $$ \mathrm{Redlich}-\mathrm{Peterson}:\ln \left(\mathrm{A}\frac{{\mathrm{C}}_{\mathrm{e}}}{{\mathrm{q}}_{\mathrm{e}}}-1\right)=\upbeta \mathrm{ln}{\mathrm{C}}_{\mathrm{e}}-\mathrm{lnB} $$

where C_e_ is the equilibrium concentration of adsorbate (mg/L) and q_0_ is the maximum monolayer coverage capacity (mg/g). K_L_ is Langmuir, K_f_ and n are Freundlich biosorption isotherm constants (L/mg), while A is Redlich-Peterson isotherm constant (L/g), B is adsorption constant (L/mg) and β is exponent, which lies between 0 and 1. The values of q_0_ and K_L_ were computed from the slope and intercept of the Langmuir plot of 1/q_e_ vs. 1/C_e_ [14]_._ In the case of the Freundlich plot of log q_e_ vs. log C_e_ allowed to obtain 1/n value from the slope and log K_f_ from intercept [21]. For Redlich-Peterson plot of ln C_e_/q_e_ vs. ln C_e_ enables the determination of Redlich-Peterson constants β and A from slope and intercept, respectively, while plotting ln (A·C_e_/q_e_-1) vs. ln C_e_ allow to obtain B from intercept [5, 22].

Thermodynamic parameters were determined using the following equations:

10 $$ {\mathrm{K}}_{\mathrm{c}}=\frac{{\mathrm{q}}_{\mathrm{e}}}{{\mathrm{C}}_{\mathrm{e}}} $$

11 $$ \Delta  \mathrm{G}{}^{\circ}=-\mathrm{RTln}{\mathrm{K}}_{\mathrm{c}} $$

12 $$ \ln {\mathrm{K}}_{\mathrm{c}}=\frac{\Delta  \mathrm{S}{}^{\circ}}{\mathrm{R}}-\frac{\Delta  \mathrm{H}{}^{\circ}}{\mathrm{R}\mathrm{T}} $$

where K_c_ is equilibrium constant, ∆G° is the change in free energy (kJ/mol), T is the temperature (K) and R is the gas constant (8.314 J/molK). ∆H° and ∆S° are enthalpy (kJ/mol) and entropy (J/mol·K) changes, which can be obtained from the slope and intercept of plots of ln K_c_ versus 1/T [7].

### Statistics and calculations

Calculations were performed with Microsoft Excel. In CCD test, coefficients of the second-order polynomial equation were calculated based on regression analysis using statistical software (STATISTICA StatSoft). The ANOVA was used to test obtained mathematical models.

## Results and discussion

In this study the role of biosorption as removal processes of two cytostatics during the fungal treatment was evaluated. The tested drugs (bleomycin and vincristine), are used together in the chemotherapy regimen for the treatment of Hodgkin’s lymphoma, but represent different groups of cytostatic drugs and have different complexity of their chemical structure. Because cytostatics might pose a risk to the environment and are not significantly removed during wastewater treatment, research on their effective elimination from the environment was necessary. The presented work involves not only fungal species most commonly used in the sorption of PhACs, represented by *T. versicolor* and *P. ostreatus*, but it also includes other less studied strains. In addition, measurements of contaminant reduction were performed across a variety of experimental conditions.

### Effect of the biomass type

Results show that removal of the assessed cytostatic drugs differed significantly, depending both on which drug was being targeted for removal, as well as whether the fungal biomass was alive or dead. Table 1 summarises the cytostatic drug sorption after 4 h.

**Table 1:** Efficiency of sorption of cytostatic drugs: bleomycin and vincristine onto white-rot fungi biomass – alive and dead (conditions: t = 4 h; C_0_ = 10 mg/L; T = 22.5 °C; fungi dose = 0.1 g_wet_/10 mL). The highest cytostatic removal efficiency by fungi in each type of experiment are underlined

	Bleomycin		Vincristine	
	Alive biomass	Dead biomass	Alive biomass	Dead biomass
*Fomes fomentarius* (CB13)	0%	38% ± 5%	7% ± 5%	13% ± 2%
*Hypholoma fasciculare* (CB15)	6% ± 11%	29% ± 12%	7% ± 6%	16% ± 4%
*Phyllotopsis nidulans* (CB14)	11% ± 6%	0%	20% ± 6%	8.6% ± 1.4%
*Pleurotus ostreatus* (BWPH)	15% ± 5%	23% ± 4%	16% ± 15%	14% ± 6%
*Trametes versicolor* (CB8)	23% ± 11%	35% ± 10%	14% ± 11%	17% ± 7%

**Fig. 1:**
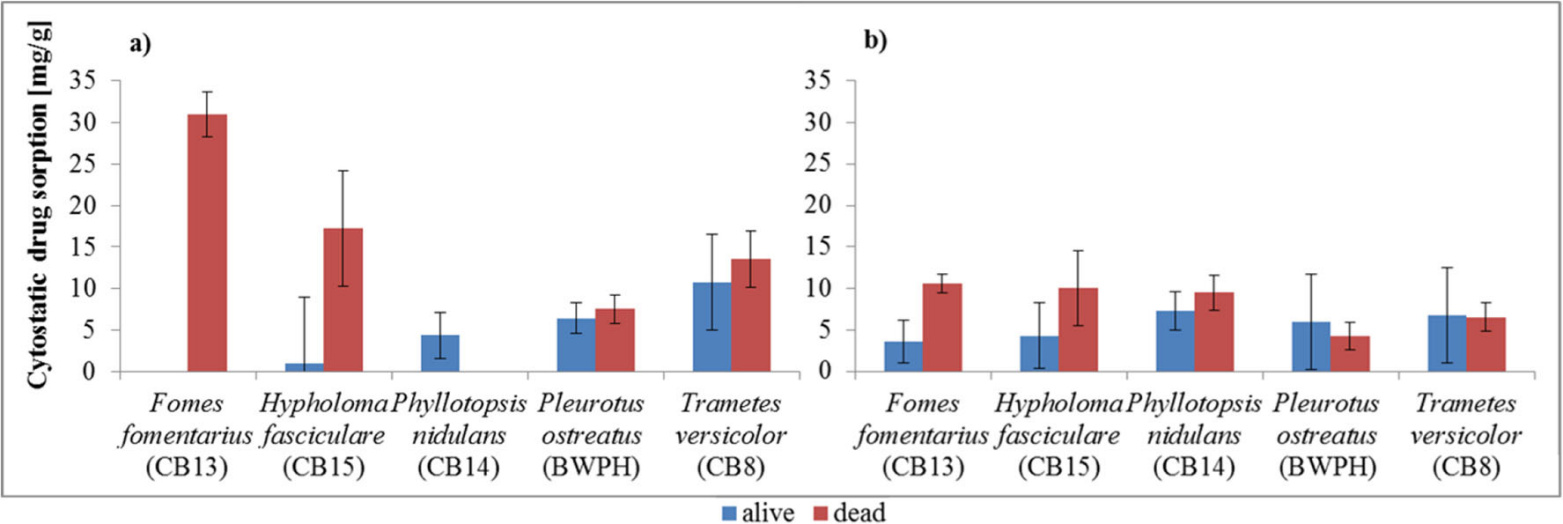
Efficiency of sorption of cytostatic drugs: (**a**) bleomycin (**b**) vincristine by 1 g of alive and dead dry fungi biomass (conditions: t = 4 h; C_0_ = 10 mg/L; T = 22.5 °C) (error bars – SD, correspond to experimental replicates)

Though the initial (wet) fungi biomass weight was 0.1 g for each test, after drying the weight varied from 0.0009 g of *P. nidulans* (CB14) to 0.0033 g of *P. ostreatus* (BWPH) in case of autoclaved specimens, and from 0.0016 g of *H. fasciculare* (CB15) to 0.0026 g of *P. nidulans* (CB14) and *P. ostreatus* (BWPH) for alive fungi (data not shown). Thus in Fig. 1 the drug removal after 4 h is calculated per 1 g of dry biomass.

In Table 1 and Fig. 1 the greatest elimination of bleomycin was in the samples with autoclaved *F. fomentarius* (CB13) and *T. versicolor* (CB8) fungal hyphae. *T. versicolor* has also the greatest removal rate for this drug among all the tested alive fungal biomasses. Visible sorption depends on the strain, which impacts specific interactions between the pharmaceutical and the surface components of each fungus. In addition, differences were also observed between sorption on alive versus dead fungal hyphae [35]. The main mechanism involved in the drug removal by alive biomass is the bioaccumulation of the compound in the cell wall and the cytoplasm, while for dead fungi the monolayer adsorption on the surface of pellets occurs mostly [32]. What is more, in active biomass, the transport in living cells may play an important role [34, 35]. Thus, the absorbed compounds can start to be biodegraded in the alive fungi by reason of intracellular enzymes [35]. In the experiment, as a safeguard against these factors, the hyphae were rinsed in water for 24 h. A drawback of using metabolically active biomass, is the possibility of suppressing toxic pollutants via cellular protective mechanisms [34].

Importantly, according to the literature, the structure of biomass (including its chemical composition), and thus its sorption capacity, can change due to the inactivation mechanism [35, 40]. These studies show that inactive (killed) biomass generally has a greater sorption ability. Past research has also shown that the fungal biomass pretreatment (for example by treating by temperature while autoclaving or by reacting with chemicals) can cause an increase in the sorption capacity [54]. Further, according to Palli et al. [51], the method of biomass inactivation can influence sorption, too. They achieved a 47% removal of diclofenac and a 15% removal of ketoprofen with heat-killed biomass of *T. versicolor*. But when NaN_3_ was used to inactivate biomass, these percentages were lower and resulted in 10% and 0%, respectively. This was likely due to the blocking of active transport across the membrane or vesicular pathways [51]. Generally, the greatest adsorption efficiency in case of heat-killed fungal pellets has been attributed to: the increase of the active sites due to the denaturation of the cell wall proteins, displaying higher biomass hydrophilicity by eliminating hydrophobic groups (e.g. diminution of −CH_3_ groups) from the cell wall and disrupting the pellet structure by expanding its porosity [32]. On the one hand, some authors believe that using dead biomass is preferable to live biomass because toxic pollutants will not have any effect on the sorption process [42]. In addition, there are no nutrition requirements to maintain the biomass growth, which makes the use of dead mycelium very interesting due to the low cost of acquisition of these biological materials, a large variety of biomasses and feasibility of its storing for long periods [19, 32, 42]. What is more, such factors as the pH value or temperature are less stringent for the biosorption carried out by dead cells and biomass can be reused for several cycles after regeneration. On the other hand, despite some advantages over live cells, dead fungal biomass, which is unable to biodegrade organic matter or to bioaccumulate nutrients, is still considered as a worse option for treating real, complex wastewater [32].

In the sorption process, the chemical character of pollutants is not without significance. The importance of the drug nature was previously confirmed by Ferrando Climent et al. [17], who conducted studies with heat-killed *T. versicolor* biomass, which demonstrated a good removal rate of tamoxifen (94% after 9 days), but no elimination of cyclophosphamide and ifosfamide. It is worth mentioning, that until now these were the only existing studies about cytostatics removal by white-rot fungi in the sorption process. Interestingly, in our experiment the reduction in the vincristine concentration was usually greater than bleomycin when alive biomass was used, while the opposite pattern was observed when dead biomass was used. In addition, in tests with dead fungal hyphae the highest vincristine elimination was shown for *T. versicolor* (CB8) and *H. fasciculare* (CB15). However, when calculated on the same dry fungi weight, the greatest elimination was shown for *F. fomentarius* (CB13) and *H. fasciculare* (CB15). In tests with alive biomass, all results indicate *P. nidulans* (CB14) as the best fungal candidate to remove vincristine. Nevertheless, the vincristine removal did not exceed 20%. Nguyen et al. [47] have reported that hydrophilic pharmaceuticals show a negligible removal rate by both active and inactivated fungi. Furthermore, studies on the elimination of TrOC (trace organic contaminants) by *T. versicolor* showed that the removal of hydrophilic compounds was negligible [47]. This indicates that the sorption process of vincristine and bleomycin as hydrophilic compounds would be low and the biotransformation by intracellular enzymes might not take place.

### Effect of the contact time

**Fig. 2:**
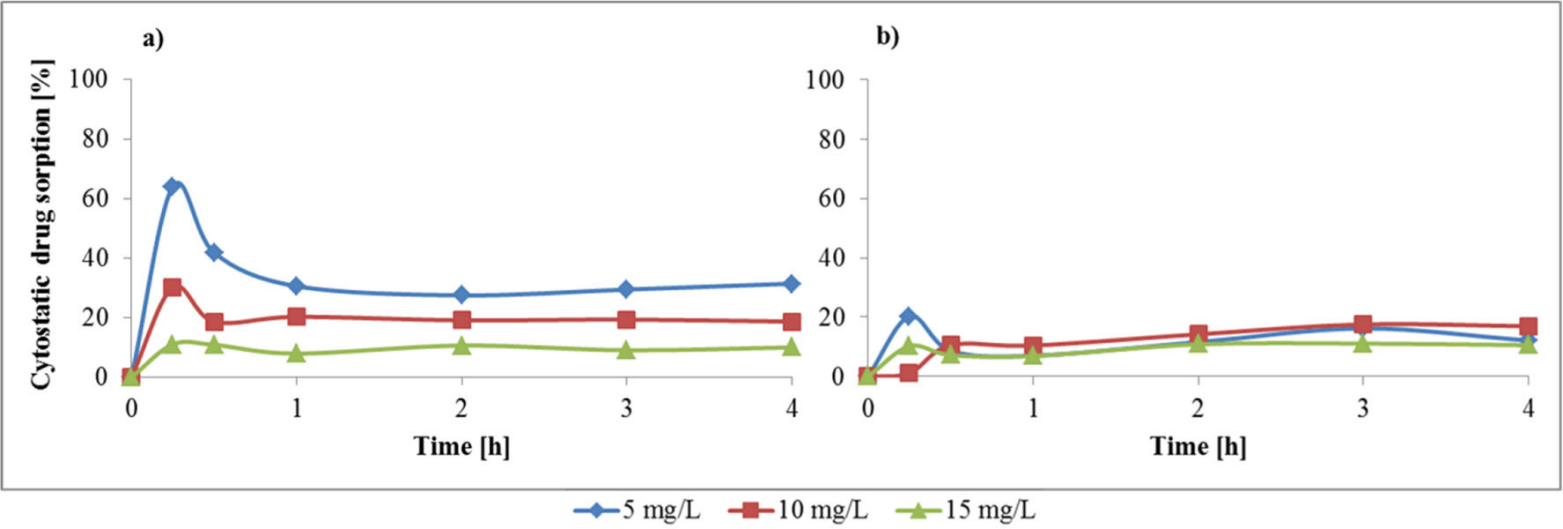
Effect of the contact time and the initial drug concentration on the sorption process of cytostatics by (**a**) *T. versicolor* (CB8) – bleomycin and (**b**) *P. nidulans* (CB14) – vincristine (conditions: T = 22.5 °C; fungi dose = 0.1 g_wet_/10 mL; fungi biomass type - alive)

According to Table 1 and Fig. 1, in tests with alive fungal biomass the greatest sorption ability was shown for *T. versicolor* (CB8), with a 23% bleomycin removal, and *P. nidulans* (CB14), with a 20% vincristine removal. Our recent results indicated that bleomycin degradation occurred after a minimum of 9 days of incubation with *T. versicolor*, while *P. nidulans* showed no biodegradation ability of vincristine [28]. It allowed us to draw a conclusion with full conviction that in the presented research the drug removal occurred in the sorption process. Because of that, further tests were conducted for these two fungi. To evaluate the influence of the contact time on the binding capacity of the drugs, biomass of both strains was exposed to aqueous drug solutions for 4 h, and measurements were made at regular intervals. The results presented in Fig. 2 show that the sorption process is fast, because the maximum removal occurred in the first two measuring points, after 15–30 min. After this initial phase the process of sorption slowed down. In the following minutes, desorption was observed, followed again by sorption until a plateau was reached. In the case of both fungi, equilibrium was reached in 2 h, with the exception of vials with 5 and 10 mg/L of vincristine, where the equilibrium was reached in 3 h.

### Effect of cytostatic the drug concentration

Four hours of sorption for drugs in the concentrations of 5 mg/L, 10 mg/L and 15 mg/L resulted in the following removal amounts respectively: 31% (equal 0.017 mg), 19% (0.023 mg) and 10% (0.017 mg) for bleomycin (Fig. 2). Similar uptake values might be attributed to the saturation of the binding sites, which are available on the sorbent [21].

In the case of vincristine, the removal amounts were 12% (0.005 mg), 17% (0.016 mg) and 11% (0.017 mg) respectively. The differences between the drug uptake from solutions in the concentration of 5 mg/L compared with the other concentrations indicates that adsorption increases with a higher initial concentration of the pharmaceutical. According to Hii et al. [21] a high concentration at the beginning of the process provided the mandatory driving force to overcome the resistance to the mass transfer of substances within the two different phases: the aqueous and the solid one. What is more, a higher concentration cause a greater number of collisions within the substance and the sorbent, which also enhances the sorption process. The amount of drug adsorbed per gram of biomass increases with higher initial concentrations of the pharmaceutical, only as long as the binding sites are not saturated [19, 21], which explains the almost equal uptake from the concentrations of 10 and 15 mg/L.

### Biosorption kinetics

**Fig. 3:**
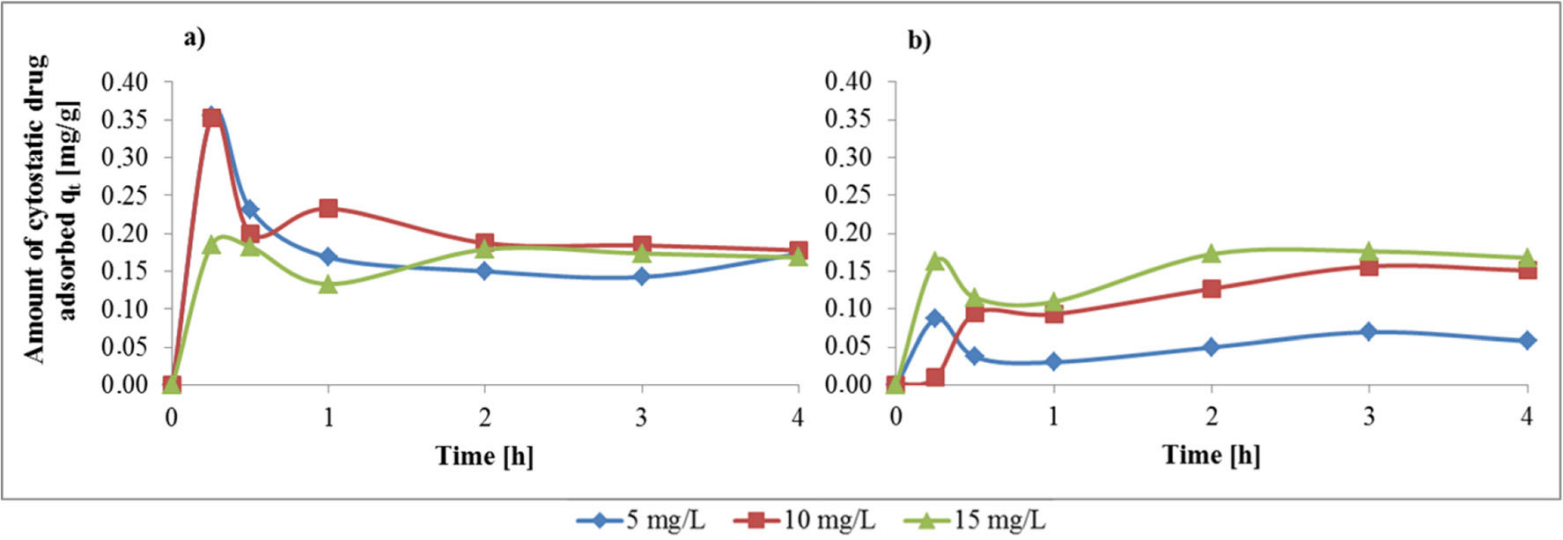
Effect of the initial cytostatics concentration on the maximum amount of drug adsorption at specific time points, conducted on (**a**) *T. versicolor* (CB8) – bleomycin and (**b**) *P. nidulans* (CB14) – vincristine (conditions: T = 22.5 °C; fungi biomass type – alive)

The adsorption kinetics results indicate that the cytostatic drug accumulation by fungi is chemical in nature, equilibrated and saturatable after 2–3 h (Fig. 3). In order to analyse the biosorption kinetics of the chosen cytostatic drugs, the pseudo-first-order, the pseudo-second-order and the Elovich kinetic models were applied to the data. The results from the above two models are presented in Table 2.

**Table 2:** Pseudo-first order, pseudo-second order and Elovich kinetic parameters for the adsorption of cytostatic drugs: bleomycin onto *T. versicolor* (CB8) and vincristine onto *P. nidulans* (CB14)

Pharmaceutic	Drug concn [mg/L]	q_e_,exp. [mg/g]	Pseudo-first order			Pseudo-second order			Elovich		
			k_1_ [1/min]	R^2^	q_e_,cal [mg/g]	k_2_ [mg/g·min]	R^2^	q_e_,cal [mg/g]	α	b	R^2^
Bleomycin	5	0.1498	0.0090	0.1585	–	−1.6680	0.9763	0.1561	−3.8·10^−5^	−15.480	0.7472
	10	0.1872	0.0295	0.3611	–	−0.6493	0.9980	0.1730	−8.5·10^−6^	−19.802	0.6647
	15	0.1789	0.0253	0.1942	–	2.2979	0.9917	0.1723	−5.1·10^−25^	−270.27	0.0441
Vincristine	5	0.0664	−0.0016	0.0069	–	0.2967	0.7946	0.0667	−1.7·10^−47^	−2000.0	0.0005
	10	0.1563	0.0051	0.0725	–	0.1375	0.9765	0.1791	0.0061	21.1864	0.8914
	15	0.1712	0.0062	0.0861	–	0.3348	0.9598	0.1713	2.6481	68.0272	0.2129

In general, a very important parameter is the initial concentration of the substance. Figure 3 shows the relation between the amount of the drug adsorbed (q_t_) vs. the duration of the experiment and the initial drug concentration. The time variation plot indicates that the removal of cytostatic drugs is rapid in initial stages, which can be related to a large concentration gradient at the beginning of the process within the sorbate and the amount of available vacant sites on the surface of the sorbent [13]. After 2–3 h, equilibrium of the process was attained. There was no significant change in time and no greater removal of cytostatics. This was also observed by Banerjee and Chattopadhyaya [7].

The tested biosorption process could be described as pseudo-second order kinetics, which is shown in Table 2. This model was well matched to the experimental data and the similarity of experimental sorption capacities and the theoretical q_e_ estimated values were observed. It suggests that especially the bleomycin sorption process was chemisorption [52]. In contrast, correlation coefficients designed in the pseudo first-order kinetic model were found to be so low that the estimation of the theoretical q_e_ values was not possible. These results indicate that the first-order kinetic model does not describe biosorption of cytostatic drugs by WRF. Also the Elovich model, which describes the second-order kinetics assuming that the actual solid surface has heterogeneous energy, exhibited lack-of-fit to the experimental data [15, 52]. The results imply that biosorption involving valence forces through the exchange or sharing of electrons between the sorbate and biosorbent might be the rate-limiting step [23].

### Biosorption isotherms

The experimental data for the removal of drugs in the sorption process (bleomycin onto *T. versicolor* (CB8) and vincristine onto *P. nidulans* (CB14)), at different concentrations, were tested with three isotherm models: Langmuir, Freundlich and Redlich-Peterson. The aim of this was to understand how the adsorbate particle was distributed between the liquid phase and the solid surface of the adsorbent at the equilibrium. The Langmuir theory assumes specific homogeneous types of adsorption within the biosorbent, meaning that the adsorbent surface is uniform with a limited number of adsorption sites, and no more adsorption takes place after the formation of a monolayer. The Freundlich isotherm model is an empirical equation, which indicates that the adsorption process takes place on a heterogeneous surface and the concentration of a drug at the equilibrium affects the adsorption capacity [7, 21, 23, 29]. The Redlich-Peterson is empirical isotherm, which mix Langmuir and Freundlich isotherms and combines elements from both equations [5].

Langmuir, Freundlich and Redlich-Peterson constant values, along with the correlation coefficients are summarised in Table 3. In the test with bleomycin the last model provided the best correlation (R^2^ = 0.98). Since this mechanism of adsorption is a mix of Langmuir and Freundlich, it does not follow ideal monolayer adsorption. At a high concentration of the adsorbate Redlich-Peterson isotherm behavior approaches that of the Freundlich. In that case A/B from Redlich-Peterson isotherm (which is 0.1217 for bleomycin), should be equal K_f_ (0.1145) of the Freundlich model, and 1-β (Redlich-Peterson exponent) should be the same as 1/n (Freundlich biosorption isotherm constant) [5]. Since those last two values are identical (0.1719), it is worth noting the significance of the 1/n constant (Freundlich model). If its value is = 1, the partition between the liquid phase and the solid one is unrelated to the concentration; when <1 it indicates a normal adsorption (chemisorption process); while >1 would indicate cooperative adsorption [14, 29]. This suggests that the bleomycin removal process by *T. versicolor* (CB8) follows chemisorption.

**Table 3:** Langmuir, Freundlich and Redlich-Peterson adsorption isotherm parameters for the sorption of cytostatic drugs: bleomycin onto *T. versicolor* (CB8) and vincristine onto *P. nidulans* (CB14)

	Langmuir			Freundlich			Redlich-Peterson			
	q_0_	K_L_	R^2^	1/n	K_f_	R^2^	β	A	B	R^2^
Bleomycin	0.2023	0.5948	0.8580	0.1719	0.1145	0.7269	0.8281	0.1143	0.9398	0.9841
Vincristine	0.6157	0.0345	0.9510	0.7062	0.0296	0.8702	0.2938	0.0295	0.9792	0.5372

In the case of vincristine, the results show that the adsorption process is defined by the Langmuir model (R^2^ = 0.95). According to that, the vincristine sorption process was found to be homogeneous. It also indicates a more physical sorption mechanism [15]. The monolayer sorption capacity of *P. nidulans* (CB14) for that drug was 0.6157 mg/g. Among used models similar adsorption prediction accuracy (Langmuir > Freundlich > Redlich-Peterson) was obtained by Román et al. [57], who also conducted research about ring structure pharmaceuticals sorption onto derived from nature sorbents.

### Effect of temperature and pH

The interactive influence of two physicochemical parameters (pH and temperature) using CCD was also tested. The experiment plan and results are presented in Table 4. The mathematical relation of the influence of independent and interactive temperature and pH on the sorption effect was approximated by the formula (polynomial quadratic), shown in the Eqs. 13 and 14. Equations were used to calculate the predicted sorption ability values.

13 $$ {\mathrm{Effect}}_{\mathrm{bleomycin}}=0.9444\ \mathrm{p}\mathrm{H}+2.5301\ \mathrm{T}-0.0533\ \mathrm{p}{\mathrm{H}}^2-0.0337\ {\mathrm{T}}^2+0.1272\ \mathrm{p}\mathrm{H}\ \mathrm{T}-16.7366 $$

14 $$ {\mathrm{Effect}}_{\mathrm{vincristine}}=-1.503\ \mathrm{p}\mathrm{H}+10.958\ \mathrm{T}+0.481\ \mathrm{p}{\mathrm{H}}^2-0.232\ {\mathrm{T}}^2-0.117\ \mathrm{p}\mathrm{H}\ \mathrm{T}-108.063 $$

**Table 4:** Central composite design experiment plan and sorption results of bleomycin onto *T. versicolor* (CB8) and vincristine onto *P. nidulans* (CB14) (T – temperature, pH, α ≈ 1.41) (conditions: t = 3 h; C_0_ = 10 mg/L; fungi dose = 0.1 g_wet_/10 mL; fungi biomass type – alive)

Experiment number		1	2	3	4	5	6	7	8	9	10	11	12
Coded values (T/pH)		0/0	-α/0	−1/−1	0/0	1/1	0/0	0/α	0/0	0/−α	1/−1	α/0	−1/1
Actual values	T [°C]	22.5	15.4	17.5	22.5	27.5	22.5	22.5	22.5	22.5	27.5	29.6	17.5
	pH	6	6	4	6	8	6	8.8	6	3.2	4	6	8
Bleomycin sorption effect [%]	Tested	41.0	28.1	34.9	28.1	54.4	51.9	58.7	55.1	27.7	48.4	55.7	35.8
	Predicted	44.0	29.7	29.0	44.0	59.5	44.0	52.5	44.0	34.8	44.3	55.0	39.2
Vincristine sorption effect [%]	Tested	13.1	3.6	8.6	12.9	7.8	14.6	26.3	13.5	8.9	9.3	0.6	11.7
	Predicted	13.5	3.2	6.1	13.5	10.9	13.5	21.9	13.5	12.7	6.6	0.5	15.0

**Fig. 4:**
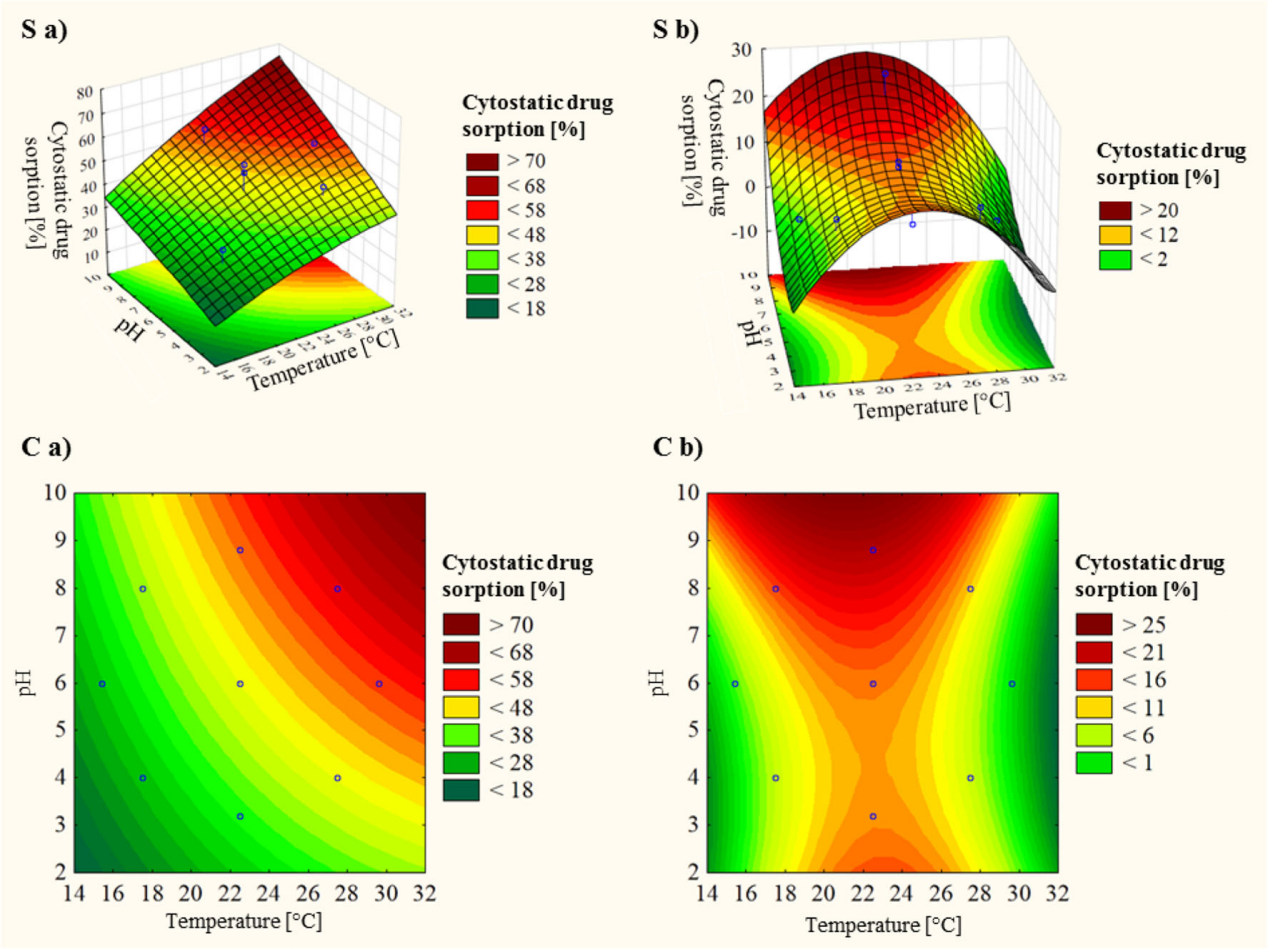
Surface (S) and contour (C) plots showing the sorption ability in the interaction effect of temperature and pH in tests conducted on (**a**) *T. versicolor* (CB8) with bleomycin and (**b**) *P. nidulans* (CB14) with vincristine (conditions: t = 3 h; C_0_ = 10 mg/L; fungi dose = 0.1 g_wet_/10 mL; fungi biomass type – alive)

The obtained models were tested using ANOVA. The bleomycin lack-of-fit test, included in the analyses, was not significant (*p* ≥ 0.05), which indicates the suitability of the presented model to describe the relationship between the tested parameters. Even though in the test with vincristine, the lack-of fit test was significant, it could be also observed significance, expressed by high Fisher (F) values and p ≤ 0.05, of pH (both linear: F = 149.9145, *p* = 0.001173 and quadratic: F = 40.3187, *p* = 0.007902) as well as quadratic temperature (F = 384.3201, *p* = 0.000290). Statistical significance was observed not only for linear temperature, but also for the interaction between the two parameters. For better visualisation of the sorption ability in relation to temperature and pH, the surface and contour plots are presented in Fig. 4.

According to our studies with bleomycin, higher temperatures result in an increased sorption capacity (Fig. 4a), which suggests that the cytostatic drug elimination process is endothermic. As stated in the literature, this is connected with an increased surface activity as well as a greater kinetic energy of molecules. The drug’s mobility increases and the retarding forces acting on diffusing the drug decrease, thereby the adsorptive capability of the adsorbent becomes greater. This effect also allows us to suppose that the mechanism of sorption is controlled by the diffusion process. Such temperature and sorption capacity dependence is also attributed to chemisorption. Because of the chemical bond between the cytostatic drug molecules and the biomass of the sorbent, the drug could not be efficiently desorbed by physical means (e.g., by shaking) [21]. In the case of vincristine, it is hard to describe the sorption process as endothermic or exothermic, as there was first an increase and then a reduction in the sorption capacity, as the temperature of the sorbate–sorbent system became higher (Fig. 4b). Even though, this test was repeated 2–3 times (each with three replicates), the results remained unchanged.

A second important parameter, which strongly influenced biosorption process was pH [23, 54]. pH alters the surface charge of the adsorbent, the degree of ionisation of the pollutant in the solution, and the structure of molecules [7]. In our study an increase in this parameter visibly caused a greater fungal biomass sorption ability (Fig. 4). The interaction between the drug and the sorbent is affected by pH. Both cytotatics (complex organic compounds) have different aromatic rings and functional groups, so they have ionisation potentials dependent on pH. This results in a net charge on the drug molecules determined by pH [21, 42]. Furthermore, many functional groups are present on the surface of the biosorbent, so the net charge on the biosorbent also depends on pH [21]. Therefore, the interaction that is observed between cytostatics and fungal biomass is connected with the ionisation states of the functional groups present at the drug molecule as well as the surface of the biosorbent. As macro fungus-based biosorbents are chemically stable in most acidic and alkaline conditions [42], reducing the efficiency of the sorption process due to biomass destruction by high or low pH is unlikely.

### Thermodynamic parameters

Thermodynamic parameters are used to describe the spontaneity and nature of the adsorption process, and the applicability of the adsorbent [15]. Thus, the change in Gibbs free energy (∆G°), enthalpy (∆H°) and entropy (∆S°) were determined for three different temperatures (15.4, 22.5, 29.6 °C) at pH 6 and the results are presented in Table 5. The positive value of ∆H° (37,630 kJ/mol) for bleomycin confirmed the endothermic nature of removal process of these drug and its chemisorption type [7], while a negative change in enthalpy for vincristine (∆H° = −233,865 kJ/mol) revealed the exothermic nature of the process for this pharmaceutical and confirmed its physisorption type [1, 15]. The positive value of ∆S° for bleomycin indicated an increase of entropy as a result of adsorption. It means that the randomness at the solid–solution interface increase during the process and the affinity of drug towards fungi biomass is good [7, 15]. In contrast, negative ∆S° for vincristine means decreased disorder. According to Banerjee and Chattopadhyaya [7] if both ∆H° and ∆S° are >0, adsorption is likely to occur spontaneously at a high temperature. Such situation occurs for the bleomycin removal by *T. versicolor* (CB8). In contrast, positive values of free energy change (ΔG°) mean that the process of drug removal was unspontaneous at all values of temperatures for bleomycin and for vincristine [18]. This surprising contradiction could be connected with the fact that – as mentioned in section 3.2 – the initial fast sorption process was then followed by desorption (and sorption again in case of vincristine) until a plateau was reached. Thus, these observed positive ΔG° values are normal as the desorption is a nonspontaneous process [43].

**Table 5:** Values of thermodynamic parameters for the removal of cytostatics: bleomycin onto *T. versicolor* (CB8) and vincristine onto *P. nidulans* (CB14)

Pharmaceutic	Temperature [K]	∆G° [kJ/mol]	∆H° [kJ/mol]	∆S° [J/mol·K]
Bleomycin	288.55	6735.41	37,630	106.79
	295.65	6227.58		
	302.75	5210.85		
Vincristine	288.55	6601.28	−233,865	−830.83
	295.65	10,239.14		
	302.75	18,472.74		

## Conclusions

In our opinion biosorption on fungal biomass is a favourable alternative to conventional processes for anticancer drug removal. This paper demonstrated that the cytostatics sorption capacity of fungi depends on such factors as the type of sorbate, sorbent and conditions of conducting the sorption process. The type of biomass connected with the species is highly important. However, the method of biomass preparation (whether biomass is alive or dead) has a great influence on the results, as well. These experiments demonstrated that inactive (killed) biomass generally has a greater sorption ability. Physico-chemical factors, like temperature and pH, were also significant, as well as the type and chemical structure of the cytostatics. The results obtained in these test will be useful in determining the role of sorption in contrast to biological degradation in the overall drug removal.
